# Changes in Milk Fat Globules and Membrane Proteins Prepared from pH-Adjusted Bovine Raw Milk

**DOI:** 10.3390/foods11244107

**Published:** 2022-12-19

**Authors:** Yanjun Sun, Yrjö H. Roos, Song Miao

**Affiliations:** 1 State Key Laboratory of Dairy Biotechnology, Shanghai Engineering Research Center of Dairy Biotechnology, Dairy Research Institute, Bright Dairy & Food Co., Ltd., Shanghai 200436, China; 2 Teagasc Food Research Centre, P61C996 Cork, Ireland; 3 School of Food and Nutritional Sciences, University College Cork, T12R229 Cork, Ireland; 4 China-Ireland International Cooperation Centre for Food Material Sciences and Structure Design, Fujian Agriculture and Forestry University, Fuzhou 350002, China

**Keywords:** milk fat globules, membrane proteins, compositions, structure

## Abstract

Milk fat globules (MFGs) have tri-layer biological membrane structures, and their compositions are gaining more interest for their physiological benefits. In this study, the changes in MFGs and milk fat globule membrane (MFGM) proteins after cream separation from different pH bovine raw milk were investigated. Raw milk samples were adjusted to pH 5.30 and 6.30 using citric acid at 25 °C. The effect of pH and centrifugation on the structure of MFGs was evaluated by means of particle size, zeta potential and confocal laser scanning microscopy (CLSM). Sodium dodecyl sulfate-polyacrylamide gel electrophoresis (SDS-PAGE) was used to analyze the proteins in the obtained fractions. It was found that both pH and centrifugation could affect the particle size of all samples. As the volume distribution (Dv; Dv (10), Dv(50)and Dv (90)) decreased, the corresponding specific surface area (SSA) increased, and span and uniformity values showed the same trend. The decrease in the zeta potential of MFG correlated with the Dv(50), which was further confirmed by CLSM observation. More butyrophilin (BTN) and periodic acid Schiff 6/7 (PAS 6/7) were lost in cream samples at pH 5.30. The findings could provide valuable knowledge for the application of MFGs ingredient in the food industry since their structures and compositions could affect their potential functional and physiological properties.

## 1. Introduction

Bovine milk is an O/W emulsion at 37 °C or above in which lipids are present in the state of microscopic globules called milk fat globules (MFGs). Approximately 95% of MFGs are comprised of heterogeneous triglycerides, which locate in the inner core of MFGs. Milk fat globule membranes (MFGMs) enclose MFGs in a three-layer biological membrane [1]. MFGMs have gained much interest due to their multilayer structure and variety amounts of polar lipids and membrane proteins. MFGM acts as a natural emulsifying agent, preventing the flocculation and coalescence of the MFGs in milk [2]. Besides, it could protect the fat against enzymatic action. 

The MFGM proteins account for approximately 1–4% of the total protein fraction in milk [1] but 25–70% of the MFGM compositions. Up-to-date proteomic techniques, including two-dimensional gel electrophoresis and mass spectrometry, revealed over 100 MFG-associated proteins [3]. Many of these proteins present at the interface of MFGM and the aqueous phase are heavily glycosylated. Most studies have focused on eight major proteins: periodic acid Schiff Ⅲ (PAS Ⅲ), butyrophilin (BTN), cluster of differentiation 36 (CD 36), mucin 1 (MUC 1), the redox enzyme xanthine oxidase/dehydrogenase (XO/XDH), lactadherin or periodic acid Schiff 6/7 (PAS 6/7), adipophilin (ADPH) and fatty acid binding protein (FABP) [1,4,5]. About 40% of the MFGM proteins are glycoproteins, and XO/XDH constitutes 12–13% of the total. Each of the other proteins in MFGM is at or below 5% [6,7].

MFGM proteins have been reviewed for their bioactivity and positive effects on defense mechanisms for breastfed infants. Research has demonstrated that some MFGM glycoproteins have been shown to protect immature infants against gut-derived pathogens [8,9]. BTN, lactadherin and MUC1 in both the human and the bovine present antimicrobial effects [10]. Defatted bovine MFGM fractions, including proteins and glycoproteins, were reported to inhibit *E. coli* O157:H7 strains adhering to HT-29 cells. Their result also showed that in vitro, breast cancer cell lines could be inhibited by FABP at extremely low concentrations [11]. MUC1 may play immune-protective roles in the suckling neonate by binding to and sequestering pathogenic microorganisms within the gut lumen. Mana et al. treated C57BL/6 mice with BTN either before or after immunization with oligodendrocyte glycoprotein. Their results showed that experimental autoimmune encephalomyelitis associated with multiple sclerosis can be prevented or suppressed by BTN [7,12].

It is assumed that the compositions and structure of MFGs change during secretion, storage, isolation and application. The MFG structure was affected by different isolation methods and conditions. Holzmüller et al. [13] reviewed the most advancing front in the MFGs composition separation and respective results. Research on the compositional and structural variation between MFGs was evaluated and consolidated. In the end, they suggested a new definition of MFGM proteins regarding the isolation method. Effect of different unit operations used in the processing on the MFGs (e.g., agitation, homogenization, heating treatment, concentration, drying and freezing.) have been investigated [2,14,15].

Acidification-induced coagulation of milk protein has been extensively used in the dairy industry. In order to reduce the loss of MFGM proteins into the casein curd, Holzmüller et al. investigated the effects of buttermilk’s pH, temperature and preheating on the rennet- and acid-induced coagulation of buttermilk [16]. According to Lopez et al., the microstructure and rheology of emulsions, as well as the stability of lipid droplets coated by MFGM, were shown to be pH-dependent [9]. MFGM-rich emulsions were prepared from butter serum using a process described in Gassi et al.’s study [17]. Under pH 5.5, lipid droplets aggregated and gels formed in the emulsions, which could affect the droplets’ microstructures [9]. 

Commercial MFGM products are usually made from dairy by-products, including whey and buttermilk. Membrane separation and cream washing are the two most typical operations in MFG compositions preparation. Membrane filtration systems are being exploited by the dairy industry for the fractionation of milk proteins. Because milk-fat-rich dairy fluid contributes to membrane fouling, skim milk is frequently utilized for membrane filtration. Higher cross-flow velocities, which produce a higher shear and transmembrane pressure (TMP) and permeate flux, could minimize fouling and prevent particle deposition. In addition, interactions between MFGM components and the size similarity with casein micelles remain problems on which some researchers are still working. Cream separator is the easiest way to separate milk into cream and skimmed milk. Before the membrane treatment, cream separation is a commonly used method to concentrate the MFGs compositions from either whey or buttermilk.

Dairy companies have been working on preparing natural MFGs and their compositions with more intact structures. Raw milk is now considering the original material to isolate MFGs because most MFGs in whey or buttermilk are fragments. The most stable and best-working pH for rennet is 5.30–6.30. Casein is a group name for the mixture of β, α and κ-casein (CN) which starts to precipitate at pH 5.30 [18]. However, just several studies are available on the effect of pH on MFGs and their membranes compositions before and after cream separation [2,19,20]. Two of the commonly used pH conditions in dairy processing, with pH values of 6.30 and 5.30, were investigated in this research. Furthermore, it is still unclear how to differentiate MFGs from MFGM damage, as well as how proteins are released during MFGM fragment disruptions.

Therefore, this study examines the changes in MFGs and MFGM proteins during cream separation from bovine milk at different pH conditions. The physicochemical properties and the microstructure of MFGs and MFGM proteins before and after cream separation are investigated. Meanwhile, the results are discussed in comparison with untreated raw milk. This study could provide technical information for a better understanding of the effect of dairy operation units on the structure and composition changes of MFGs and MFGMs. This is crucial for future studies on the physiological properties of MFGs and related compositions. 

## 2. Materials and Methods

### 2.1. Materials

Raw bovine milk (fat 4.08 ± 0.02%, protein 3.57 ± 0.01%, dry matter 13.03 ± 0.02%, pH 6.74 ± 0.04) was collected from the Teagasc dairy herd (Moorepark, Fermoy, Co. Cork, Ireland). The raw bovine milk was untreated by any other unit operation except for the rapid chilling from 37 to 4 °C with a plate heat exchanger (UK Exchangers Ltd., Buckinghamshire, UK). 

Chemicals for gel electrophoresis were bought from Thermo Scientific Inc. (Thermo Fisher Scientific Inc., Cork, Ireland). All solutions were prepared using MilliQ water except as otherwise noted.

### 2.2. Preparation of Samples 

Centrifugal separation was performed using a disk bowl centrifugal separator (FT 15 Disc Bowl Centrifuge, Armfield co., Ringwood, UK) equipped with 19 disks. Firstly, 3 batches of raw milk collected from a 4 °C refrigerator, each of 5 kg, were preheated to 25–30 °C in a water bath (GFL 1012, Gesellschaft fuer Labortec, Burgwedel, Germany). Before the raw milk was separated into cream and skim fractions by a centrifugal separator, 2 batches of 5 kg raw milk were adjusted to pH 5.30 and 6.30, respectively. Batches of pH 6.74 ± 0.04 were taken as the control reference. Secondly, we continued to heat all batches of raw milk to 50–55 °C and incubated them during the whole separation process. The skim milk phase was added back into the separator one more time to maximally defat the skim milk. The cream and skim milk phases of each batch were collected separately and kept at 4 °C before analysis. The collected samples were named 6.74 R (raw milk pH 6.74), 6.30 R (raw milk pH 6.30), 5.30 R (raw milk pH 5.30), 6.74 C (cream pH 6.74), 6.30 C (cream pH 6.30), 5.30 C (cream pH 5.30), 6.74 S (skim milk pH 6.74), 6.30 S (skim milk pH 6.30) and 5.30 S (skim milk pH 5.30). Throughout the heating and pH adjustment process, all batches of samples were stirred gently to avoid part denaturation of the proteins induced by uneven temperature and excessive acidification, and 0.02% (*g*/*g*) azide sodium was added to prevent microbiological growth. 

### 2.3. Compositions Analysis

Fat, protein and non-fat solids (SNFs) were analyzed with DairySpec FT-15 (Bentley Instruments Inc., Chaska, MN, USA). 30 mL of liquid sample was mixed thoroughly before analysis to avoid uneven sampling. All samples from the 4 °C storage were preheated to 25 °C in a water bath. For fat content analysis, cream samples needed to be diluted 10 times with MilliQ water. After the dilution of cream samples, the protein content is close to the detection limit (0-50%)of DairySpec FT-15, so that Kjeldahl method [21] was used to quantitatively analyze the protein content of cream samples (N × 6.38). 

### 2.4. Particle Size Analysis

Particle size distributions of different pH samples were measured using a Mastersizer 3000 (Malvern Instruments, Malvern, UK) equipped with a laser light scattering. The refractive index for milk fat and water was set to 1.456 and 1.333, respectively. Particle sizes were recorded after at least 30 s of stabilization after adding samples to the measurement device. The amount of samples depends on if the obscuration could reach the range of 3–20% [4,22]. The data were calculated according to Mie theory. 

Samples were analyzed 3 times, and the data are expressed as volume distributions. Dv (10) and Dv (90) stand for the size of particles below which 10%/90% of the sample lies. Dv (50) means the size in microns at which 50% of the sample is smaller and 50% is larger, also known as the median of the volume distribution. Span is the measurement of the width of the distribution. The narrower the distribution, the smaller the span becomes. Uniformity is a measure of the absolute deviation from the median. Uniformity could demonstrate the degree of particle size distribution and the gradient of the distribution curve [23]. The value of the specific surface area (SSA) of all samples was calculated as the surface area of the particles per unit weight. The equation is as follows:

(1) SSA=6/pD [3,2]

where p is the particle density, and D [3,2] is the surface-weighted mean.

### 2.5. Zeta Potential 

The zeta potential, as an indicator for the change of the milk fat globule surface, was analyzed by a dynamic light scattering instrument (Nano-ZS, Malvern Instruments Ltd., Worcestershire, UK) [22]. The Zetasizer software version 7.12 (Malvern Instruments Ltd.) was used to collect and analyze data. Zeta potential is evaluated by measuring the electrophoresis mobility and then calculated by the Henry equation. The Henry equation is:

(2) $$U_{E}=2\varepsilon zf\left(\mathrm{Ka}\right)/3\eta$$

where:

z: Zeta potential

$U_{E}$: Electrophoretic mobility

ε: Dielectric constant

η: Viscosity

*f*(Ka): Henry’s function, in this case, is 1.5 and is referred to as the Smoluchowski approximation since the double layer κ-1 of MFGs (around 1.1 nm) is smaller than their radius [24]. 

The electrophoretic mobility is obtained by performing an electrophoresis experiment on the sample and measuring the velocity of the particles using laser Doppler velocimetry. Samples were diluted by MilliQ water and analyzed in a Malvern Dip Cell at 25 °C. The requirement that zeta potential tests should be performed on diluted dispersion, which modifies the samples’ structural characteristics, is a drawback. Additionally, dilution times and diluent types have a significant impact on the outcomes. In order to reduce the diluent-type effect, MilliQ water was used as the diluent. In preliminary experiments, the effect of MilliQ water dilution times (50, 100 and 1000 times) on the zeta potential was tested, and there was no significant difference between 100 and 1000 times. Thus, all samples were diluted 100 times in this research, and a 2-min equilibrium time was allowed before each measurement. Three readings were collected from each sample.

### 2.6. Sodium Dodecyl Sulphate Polyacrylamide Gel Electrophoresis (SDS–PAGE)

SDS-PAGE was conducted according to the method described by Ye, A. et al. with revised [25].

A Mini-PROTEIN Tetra cell was used for the protein analysis (Bio-Rad, Laboratories, inc. USA). Proteins in the raw milk, skim milk and cream were analyzed by reducing SDS–PAGE. Under reducing conditions, both non-covalently-linked and disulfide-linked complexes were dispersed and migrated into the resolving gel. The NuPAGE MOPS SDS Running Buffer (20×; 50 mM MOPS, 50 mM Tris Base, 0.1% SDS, 1 mM EDTA, pH 7.7) was purchased from Thermo Fisher Scientific Inc., which is used with NuPAGE Novex Bis-Tris Gels (Thermo Fisher Scientific Inc., Cork, Ireland) to resolve mid-size proteins. For electrophoresis, dilute this buffer to 1× with MilliQ water. The NuPAGE Antioxidant (Thermo Fisher Scientific Inc., Cork Ireland) was used to avoid reoxidization.

MilliQ water was used to dilute the samples to a protein concentration of 40 µg/mL, then mixed with 4× NuPAGE LDS sample buffer (106 mM Tris–HCl, 141 mM Tris Base, 2% LDS, 10% glycerol, 0.51 mM EDTA, 0.22 mM SERVA coomassie blue G250, 0.175 mM phenol red, pH 8.5). The NuPAGE Reducing Agent (10×; Thermo Fisher Scientific Inc., Cork, Ireland) contained 500 mM dithiothreitol (DTT) and was added to the samples at least one hour before loading the samples to prevent reoxidation during storage and producing inconsistent results. 

A criterion 12% NuPAGE Novex Bis-Tris Gels (Thermo Fisher Scientific Inc., Cork, Ireland) was used to separate proteins. All samples were heated to 70 °C and inoculated for 5 min to denature the proteins. Before being loaded into the gels, denatured samples needed to cool down to room temperature and centrifuged. A marker with a molecular weight between 10–200 kDa (Thermo Fisher Scientific Inc., Ireland) was used. 

Gels were run at 120 V for 45 min and then dyed the proteins with Coomassie blue for more than 60 min with gentle shaking. A solution containing 45% ethanol and 5% acetic acid was used to destain the gels. After being destained for 50 min, the solution was changed to remove the stain overnight. Typical MFGM proteins (PAS 6/7, BTN and XO) and milk serum proteins (α-LA, β-LG and caseins) were selected to relatively quantify the analysis. The relative density of representative proteins was analyzed using ImageJ software 1.36 b (Wayne Rasband, National Institutes of Health, Bethesda, MD, USA) and normalized to 100%. 

### 2.7. Confocal Laser Scanning Microscopy (CLSM)

CLSM could be used to qualitatively analyze different types of proteins adsorbed on the surface of MFGs, which demonstrates the effect of treatment on the reconstruction of protein and fat globules. Non-polar lipids, which were mostly triglyceride, were labeled with Nile Red (9- (diethylamino)-5 H-benzo[*a*]phenoxazin-5-one, Sigma–Aldrich^®^, Merck Life Science Ltd., Cork, Ireland), and serum protein and glycoproteins with fluorescent Fast Green (FCF; N-ethyl-N-[4-[[4-[ethyl[(3-sulfophenyl)methyl]amino]phenyl](4-hydroxy-2-sulfophenyl)methylene]-2,5-cyclohexadien-1-ylidene]-3-sulfo-benzenemethanaminium, disodium salt, Cayman Chemical, Ann Arbor, MI, USA) and wheat germ agglutinin (WGA; Alexa Fluro^®^ 488 conjugate, Invitrogen, Ltd., Renfrew, UK), respectively [26,27]. Considering the low fat content of the skim milk, only the raw milk and cream samples (80 μL) were dual-stained with a 10 µL mixture of Nile red (0.1%, *w*/*v*, in polyethylene glycol-200) and FCF (0.1%, w/v, in distilled water; FCF: Nile red = 3:1 *v*/*v*). In addition, WGA (50 μL, 1 mg/mL in PBS) was mixed with Nile red in a proportion of 1:1 (*v*/*v*). All dyed samples were kept in the dark for 30 min at room temperature to ensure complete staining. The proportion between the sample and stain was optimized in the pre-experiment to take account of the variation of pH and incomplete dyeing.

Leica TCS SP5 microscope (Leica Microsystems GmbH, Wetzlar, Germany) was used to detect at room temperature (25 ± 2 °C). Nile red, FCF and WGA were excited using a He-Ne laser, and emission wavelengths were detected between 565–625 nm, 632–719 nm and 493–550 nm, respectively. A 63×oil-immersion lens was used to observe the samples. Two channels—FCF or WGA channel 1 and Nile red channel 2—were used for CLSM observation. Dual-stain imageswere obtained by merging images produced in 2 different channels. No less than 10 images were recorded for each sample. Of all the images, the representative 1 for each experiment was chosen. Leica application suite X (3.7.5.24914; Leica Microsystem CMS GmbH, Wetzlar, Germany) was used for image processing and analysis. Two-dimensional images presented a resolution of 512 × 512 pixels, and a scaling factor was used to convert the pixel scale values into micrometers. 

### 2.8. Statistical Analysis

There was triplication of each experiment. Each value represents the mean of at least 3 measurements, and values are expressed as means ± standard deviations. A 1-way ANOVA was used to calculate significant differences (95.0% confidence level) between the samples using Minitab 17 Statistical Software (Minitab Inc. State College, PA, USA).

## 3. Results

### 3.1. Composition Analysis

The fat, protein and SNF contents of all samples are shown in Table 1. The amount of protein included serum proteins and MFGM proteins. After cream separation, the fat in all cream samples was between 39.90–40.5% ignoring the pH variation, but the protein content significantly varied with different pH values. 6.30 C had the most proteins (2.01 ± 0.02%), followed by 5.30 C and 6.74 C (1.76 ± 0.03% and 1.50 ± 0.01%). There was no significant difference in the amount of fat, proteins and SNF in raw milk samples (*p* > 0.05) as a function of pH decrease. For skim milk samples, 6.74 S and 6.30 S presented a similar protein amount, while 5.30 S had a higher content of fat and protein. From the data analysis above, we could speculate that the pH 5.30 condition was the main reason for the difference. The pH 5.3 condition is close to the pI of some proteins. Partial aggregates of proteins in raw milk are easy to get into the skim milk phase during the cream separation process.

### 3.2. Particle Size

The Dv(10), Dv(50) and Dv(90) of cream samples are significantly higher than those of raw milk and skim milk samples (*p* < 0.05) except for skim milk under the pH 5.3 condition (Table 2). This result could be explained by the fact that the fat globules present bigger particle sizes than milk proteins. According to the results of composition analysis in Section 3.1, fat content in cream samples was significantly higher than that in raw milk and skim milk samples. It was reported that the size of milk fat globules in untreated milk varied from 0.2–15 μm, with a mean diameter of 3–4 μm [28]. Native casein micelles which exist as the particles in milk, consist of casein proteins and calcium phosphate [18]. 

Native bovine milk is a semi-stable emulsion at 37 °C or above in which casein micelles at pH 6.70 are generally assumed to remain in suspension due to the hairy layer of κ-casein providing steric and electrostatic repulsion [29]. Hydrophobic and hydrophilic radicals on the surface of MFGs, acting as natural emulsifying agents, play an important role in preventing the flocculation and coalescence of the globules and keeping the charge balance between milk protein and fat globules. Acid induced an increased attachment of milk protein to the surface of MFGs due to the charge change of MFGs and serum protein. This could be the reason for the slight increase of Dv(10), Dv(50) and Dv(90) in raw milk samples with the pH decrease. Cream samples presented the opposite trend to raw milk with the smallest Dv(10), Dv(50) and Dv(90) at pH 5.30, which were between 0.97 ± 0.04 and 48.14 ± 8.50 μm. This is inconsistent with Holzmüller et al. [16], who reported larger particles with decreasing pH. The shear force in the disc stack of the cream separator resulted in MFGs disruption, which led to the coalescence of the fat globules and then contributed to the increase of particle size [30]. 

**Figure 1:**
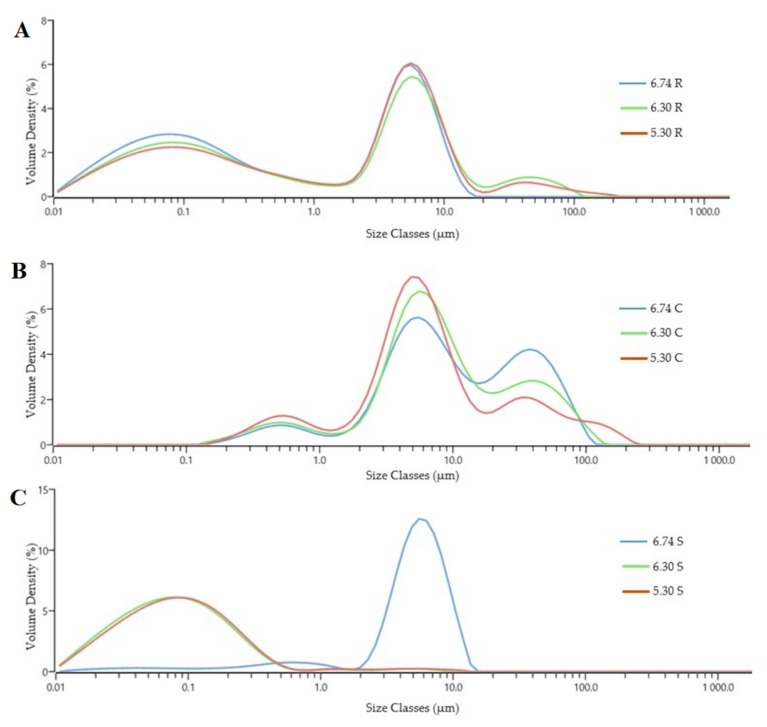
Volume density (%) of raw milk (**A**), cream samples (**B**) and skim milk (**C**) at different pH. 6.74 R (raw milk pH 6.74), 6.30 R (raw milk pH 6.30), 5.30 R (raw milk pH 5.30), 6.74 C (cream pH 6.74), 6.30 C (cream pH 6.30), 5.30 C (cream pH 5.30), 6.74 S (skim milk pH 6.74), 6.30 S (skim milk pH 6.30) and 5.30 S (skim milk pH 5.30).

No effect of pH on Dv(10), Dv(50) and Dv(90) of skim milk samples was detected between 6.74 and 6.30, but there was a sharp increase as the pH continually decreased to 5.30. Casein, with an isoelectric point at pH 4.6, tended to coalesce and precipitate from milk with lower pH, at which condition particle size values moved towards large ones (Table 2 and Figure 1C). 

Data in Table 2 showed that as the Dv(10), Dv(50) and Dv(90) decreased, the corresponding SSA increased. This accords with P. Sharma et al.’s report, which showed that the D [3,2] of untreated whole milk decreased with the intensity of pulsed electric field treatment [19], while the corresponding SSA increased. The trends for span and uniformity were comparable. It was shown that more homogenous particle sizes in samples presented smaller uniformity [23]. Cream samples presented higher span and uniformity values than raw milk and skim milk samples. Accordingly, particle size in cream samples was wider and more multitudinous (Figure 1B). Generally, the main particle size of raw milk samples was between 0.01 and 174 μm (Figure 1A), while skim milk samples with pH 6.74 and 6.30 present the narrowest size distribution, between 0.01 and 9.98 μm (Figure 1C). 

According to these results, we speculated that under the same pH condition, the change in particle size of fat globules could be attributed to the shear force produced by the separator. It could be due to the coalescence or damage of MFGs and the loss of MFGM materials [31]. Besides, most of the presumed stabilizing materials (caseins and whey proteins) are lost during the cream separation. However, in this and Holzmüller et al.’s [4] previous research, the shear force of the cream separator was not quantitatively analyzed, and the relation between the extent of shear force and fat globules damage was not confirmed. Thus, the effect of shear force on the integrity of MFGs still needs to be further investigated. In addition, we could conclude that the pH variation could be an important factor in changing the particle size of all samples. Either because the change of proteins on the surface of MFGs, or the attachment of casein or whey proteins on MFGMs. 

### 3.3. Zeta Potential 

**Figure 2:**
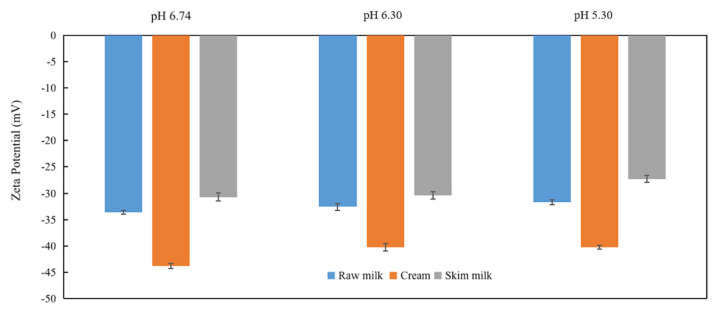
Zeta potential of raw milk, cream and skim milk as a function of pH.

The stability of casein micelles and milk fat has been associated with zeta potential value. The measured zeta potentials of all samples are illustrated in Figure 2. All of the samples showed a negative zeta potential. According to Michalski et al. [32], casein micelles and MFGs both have negative charges between pH 6.60–6.70 at 25 °C, and their apparent zeta potential is negative. Thus, it should be noted that the description of the increase or decrease of zeta potential in this study is not used algebraically but refer to their absolute values. Hence, it should be understood that algebra is not used in this research to describe how the zeta potential increases or decreases; rather, it refers to the absolute magnitude of the zeta potential.

The zeta potential of raw milk with pH 6.74, when measured at 25 °C, was −33.60 mV (Figure 2), which was much higher than the previously published reports. P. Sharma et al. [19] and Michalski et al. [33] showed that the zeta potential value of whole milk at physiological pH (6.60–7.0) was −13.7 ± 0.1 mV. Some other researchers published −22 mV for whole milk at pH 6.67 [34,35]. Jukkola et al. investigated the effect of diafiltration media on the separation of MFGs via microfiltration [20]. The zeta potential values for the feed milk were −30 mV, which is quite close to our results. Comparing zeta potential results of different research is challenging since many factors greatly influence the obtained value. Michalski, Michel et al. [33] found that different analysis techniques, including laser Doppler electrophoresis (particularly the scattered light angle) or electroacoustic methods, could affect the zeta potential [19]. Other experimental conditions (e.g., diluent and temperature) could also significantly affect the zeta potential value [36] and hence the zeta potential. There was no significant difference in the zeta potential of raw milk between pH 6.74 and 5.30 (25 °C; *p* > 0.05) 

The zeta potential of skim samples of different pH varied from −27.3 to −30.7 mV, which was relatively lower than that of cream and raw milk samples. At a pH of 6.7–6.8 (25 °C), reconstituted skim milk was found to have a zeta potential of −34.6 ± 3.6 mV [37]. The charge of casein switches from negative to positive as the pH decrease [38]. This could explain why the zeta potential of skim milk samples at pH 5.30 (−27.3 mV) was significantly (*p* < 0.05) lower than that of pH 6.74 (−30.7 mV). At lower pH, there was more adsorption of caseins and whey proteins on the MFGs surface in raw milk samples. However, after mechanical treatment in the centrifugation process, the amount of the adsorbing serum proteins decreased, thus decreasing the zeta potential in skim samples. 

pH 5.30 condition further decreased the zeta potential of cream samples to −40.3 mV. This might be a result of a modification to the MFGM protein, which leads to an increase in the exposure of negatively charged and/or hydrophobic areas [37]. The findings for zeta potential were consistent with those for particle size at pH 5.30, leading to the smaller particle size of cream samples. For conducting particles, the zeta potential value generally depends on their particle size [33,39]. 

### 3.4. SDS-PAGE 

**Figure 3:**
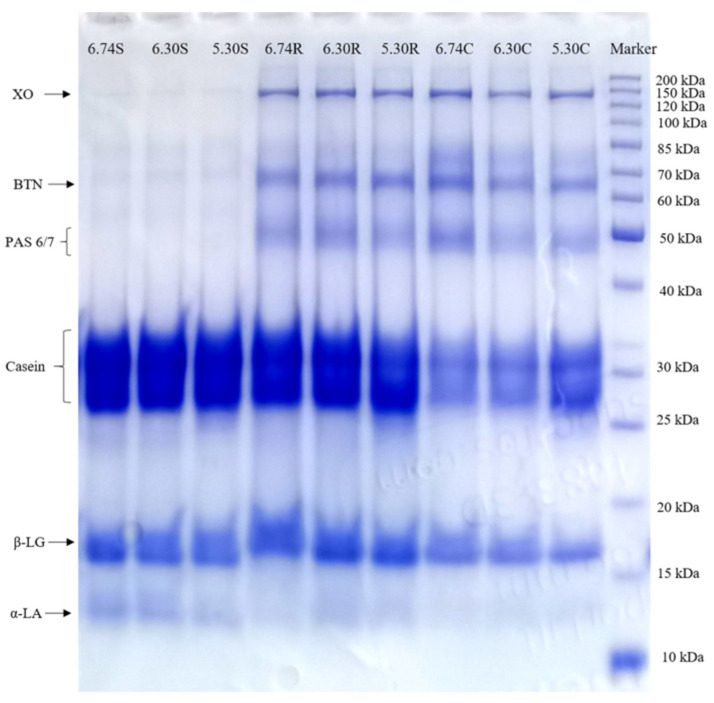
SDS-PAGE shows the predominant proteins presenting in raw milk, cream and skim milk with different pH.^.^ 6.74 R (raw milk pH 6.74), 6.30 R (raw milk pH 6.30), 5.30 R (raw milk pH 5.30), 6.74 C (cream pH 6.74), 6.30 C (cream pH 6.30), 5.30 C (cream pH 5.30), 6.74 S (skim milk pH 6.74), 6.30 S (skim milk pH 6.30) and 5.30 S (skim milk pH 5.30). PAS 6/7, periodic acid Schiff 6 and 7; BTN, butyrophilin; XO, xanthine oxidase; α-LA, α-lactalbumin; β-LG, β-lactoglobulin.

**Figure 4:**
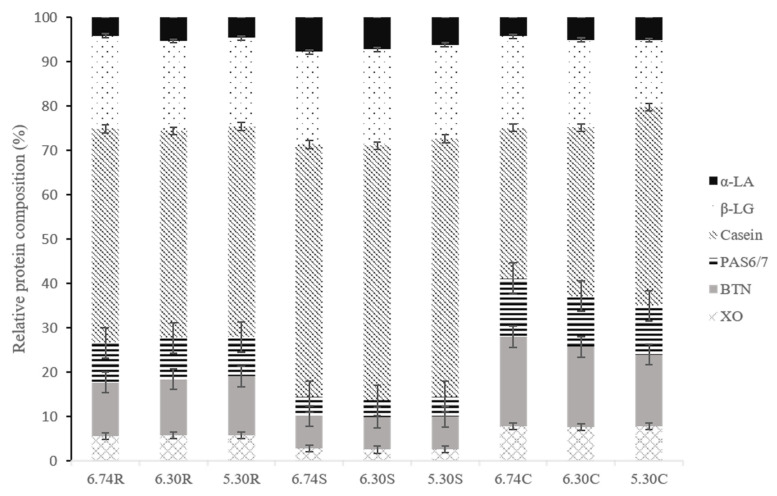
Relative protein compositions of raw milk, cream and skim milk samples derived from densitometry measurements of bands in a representative SDS-PAGE gel; 6.74 R (raw milk pH 6.74), 6.30 R (raw milk pH 6.30), 5.30 R (raw milk pH 5.30), 6.74 C (cream pH 6.74), 6.30 C (cream pH 6.30), 5.30 C (cream pH 5.30), 6.74 S (skim milk pH 6.74), 6.30 S (skim milk pH 6.30) and 5.30 S (skim milk pH 5.30).

The change in serum and MFGM protein with the pH variation was determined by SDS-PAGE under reducing condition (Figure 3). Meanwhile, relative protein content was quantified by combining densitometry with the total lane signal intensity (Figure 4). The electrophoresis mobility of proteins was used to define their molecular weight as well as numerous published literatures: caseins 19–34 kDa, β-LG 18 kDa, α-LA 14 kDa, XO 146–155 kDa, BTN 66–67 kDa and PAS 6/7 47–59 kDa [5,40,41,42,43].

Qualitative differences between the individual samples depend on the intensity of the protein bands. Higher relative intensities typically denote a higher protein content. A loss of XO, BTN and PAS 6/7 in skim milk samples was observed in Figure 3. The protein profile of cream samples differed from that of the skim milk, with more dominant MFGM proteins at the expense of casein, β-LG and α-LA. 

It was more prominent in the incorporation of α-LA in cream samples at lower pH during centrifugal separation. The relative content of α -LA in cream samples at pH 5.30 increased by 19.18% than that in 6.74 C samples (Figure 4). However, at pH 5.30, the levels of α-LA in both the raw milk and the skim samples were reduced, which was predicted based on the influence of charge on the solubility of α-LA in casein micelles. The solubility of α-LA decreased by a higher calcium quantity in the aqueous phase caused by the release of calcium phosphate from micelles under acidic conditions [44].

β-LG was reported to interchangeably interact with MFGM proteins via sulfhydryl-disulfide, then bound to the casein micelles through κ-casein [25]. A lower pH condition apparently is not helpful for this interaction since either raw milk or cream samples showed less β-LG amounts at pH 5.30. 

Regarding BTN, PAS 6/7 and XO, both Figure 3 and Figure 4 showed the lower pH led to a higher BTN and PAS 6/7 loss in cream samples. However, the relative content of XO in all samples had no significant variation as pH decreased (*p* > 0.05). The theoretical pI of BTN is 5.32. [16,40], this protein tends to precipitate at pH 5.30. This could explain the more intense BTN bands in raw milk samples at pH 5.30 since BTN was released from the MFGM and formed purer aggregates. BTN is typically referred to as an integral protein, whereas PAS 6/7 is classified as a periphery protein [4]. BTN and part of XO were reported to strongly attach to MFGM after the extracted pre-treatment with Triton X-100, sodium deoxycholate and guanidine hydrochloride [45]. The amount of PAS 6/7 in the 6.74 S samples was reduced by 51.90% more than that in the 6.74 R samples, with a relative intensity of only 7.34 and 4.29% for BTN and XO in 6.74 S, respectively (Figure 4). This outcome was inconsistent with the binding of MFGM proteins previously mentioned. It was positive to find that cream samples contained a more significant amount of MFGM proteins than skim and raw milk samples (*p* < 0.05), with a range of 7.59–88%, 16.09–20.12% and 10.98–13.14 %, respectively. This confirmed that not only loosely-bound membrane components but also integral proteins were lost during cream separation [46]. The results above are consistent with the particle size shift in Section 3.2, which shows that cream samples present higher Dv (10), Dv (50) and Dv (90) values than skim and raw milk.

Overall, shear force plus the pH decreasing during the cream separation process did change the protein compositions of MFGM. Probably due to fat globule damage, parts of MFGM materials are released from the fat globule and partitioned into the serum phase or associated with the serum proteins.

### 3.5. CLSM 

The CLSM technique was developed to detect the MFGs in their native or specific environments (i.e., certain pH conditions) in a non-destructive manner. In our research, the samples were dual-stained by combining Nile Red with FCF or WGA.

**Figure 5:**
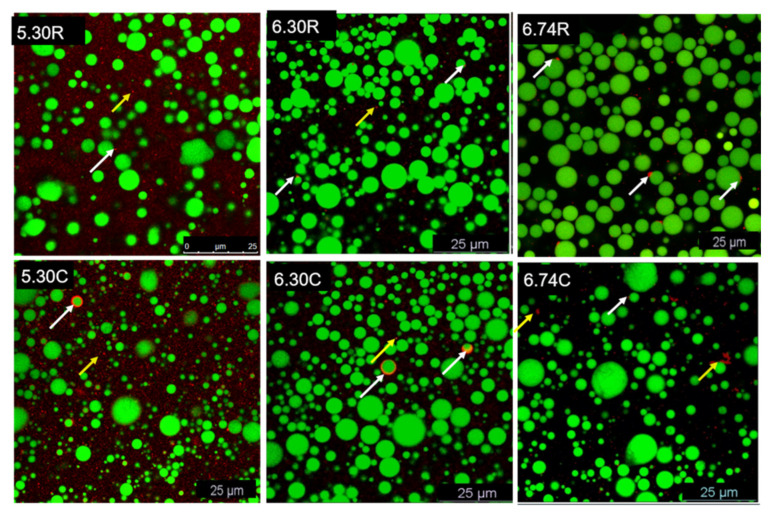
Confocal images of dual-stained milk fat globules in raw milk and cream at different pH. White and yellow arrows mark the serum proteins attaching on the surface of MFGs and drifting in the liquid phase, respectively. MFGs stained with Nile Red (green), and serum proteins stained with fluorescent Fast Green. 6.74 R (raw milk pH 6.74), 6.30 R (raw milk pH 6.30), 5.30 R (raw milk pH 5.30), 6.74 C (cream pH 6.74), 6.30 C (cream pH 6.30), 5.30 C (cream pH 5.30), 6.74 S (skim milk pH 6.74), 6.30 S (skim milk pH 6.30) and 5.30 S (skim milk pH 5.30).

**Figure 6:**
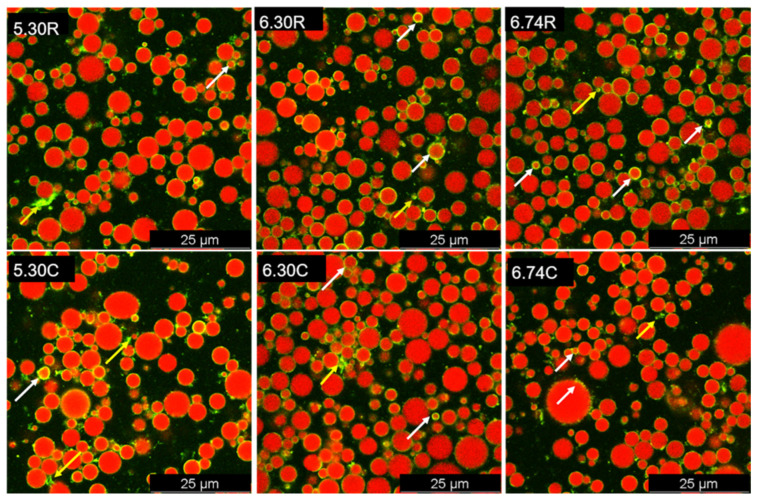
Confocal micrographs of double-stained milk fat globules in raw milk and cream at different pH. White arrows mark the glycoproteins attaching to the surface of MFGs, while yellow arrows mark the coalescent glycoproteins. MFGs were dyed with Nile Red (red), and glycoproteins were dyed with wheat germ agglutinin (WGA; yellow-green. 6.74 R (raw milk pH 6.74), 6.30 R (raw milk pH 6.30), 5.30 R (raw milk pH 5.30), 6.74 C (cream pH 6.74), 6.30 C (cream pH 6.30), 5.30 C (cream pH 5.30), 6.74 S (skim milk pH 6.74), 6.30 S (skim milk pH 6.30) and 5.30 S (skim milk pH 5.30).

The microstructural comparison of raw milk and cream samples with different pH are shown in Figure 5 and Figure 6. Just as the conclusions from amounts of previous research, milk fat existed in the state of regular spheres [27,47,48]. Triglyceride was the main composition of MFGs and could be observed in the core of MFGs, which showed green (Figure 5) or red fluorescence (Figure 6). Serum proteins including casein and whey protein were stained using FCF and presented red color, as shown in Figure 5. MFGs were covered by MFGM that consisted of heterogeneous glycoproteins stained by fluorescent green color (Figure 6).

Cream samples contained fewer serum proteins than raw milk samples, which was confirmed by the weaker fluorescent signal (red color). This could be identified by the results of the composition analysis in Section 3.1. Serum proteins, especially caseins, tended to form network structures at pH 5.30. In comparison with pH 6.30 and 6.74, either 5.30 R or 5.30 C samples showed the evident network structure formed by serum proteins (Figure 5 and Figure 6. Cream samples showed bigger MFGs than raw milk samples, and this result is in accordance with the particle size analysis in Section 3.2. 

CLSM images demonstrated that pH adjustment promoted serum protein adsorption on the outer layer of fat globules (Figure 5). Compared to cream samples, protein adsorption was more pronounced in raw milk samples, perhaps due to the lower protein content of cream samples or the desorption from the surface of MFGs during centrifugation. As for the glycoproteins, the fluorescent signals on the surface of cream samples were more discontinuous than that for the raw milk samples (Figure 6). Meanwhile, at pH 5.30, more flocculation and coalescence of MFGM proteins were observed on the surface or in the serum phase of raw milk and cream samples. This result is in contrast to the SDS-PAGE analysis that showed the more amounts of MFGM proteins in cream samples and lower pH value further exacerbated this phenomenon. We speculated that SDS-PAGE samples preparation in this research could not tell the exact location of MFGM proteins which were either on the surface of the MFGs or in the serum phase. Since the CLSM is only a qualitative analysis method, it is impossible to accurately calculate the total MFGM proteins in the observing views. Thus, non-invasive and quantitative methods are expected to be developed in further research. 

## 4. Conclusions

In spite of the functionality and physiology of MFGs being widely researched in recent years, the effect of dairy processing on this is poorly understood. This research unprecedentedly demonstrated the implication of pH adjusting on the structure of MFGs and MFGM protein compositions during the cream separation. SDS-PAGE images and relative quantitative data showed that a loss of MFGM proteins after cream separation was inevitable, especially the BTN and PAS 6/7 at pH 5.30. Meanwhile, casein and whey protein tended to absorb on the surface of MFGs or form networks under the pH 5.30 condition, but mechanical force during centrifugation could desorb the attached serum proteins or the intrinsic glycoproteins. Thus, this led to the particle size change of MFGs and the rearrangement of MFGM compositions. Particle size (Dv (10), Dv(50) and Dv (90)) and zeta potential of cream samples decrease at lower pH 5.30.

Only two typical pH conditions (pH 5.30 and 6.30) were investigated in the present study, and how the pH variation and centrifugation affected the lipids of MFGMs and their physiological properties was not studied. Considering some analysis methods probably bringing about the structure or compositional changes of MFGs, more non-invasive methods need to be developed to conduct the real in situ observation.

## Figures and Tables

**Table 1:** Composition analysis of raw milk, skim milk and cream samples at different pH.

Samples	Fat (g/100 g)	Protein (g/100 g)	SNF (Solid Non-Fat; g/100 g)
6.74 R	4.85 ± 0.04 ^a^	3.55 ± 0.00 ^a^	12.36 ± 0.10 ^a^
6.30 R	4.83 ± 0.02 ^a^	3.66 ± 0.04 ^a^	12.33 ± 0.12 ^a^
5.30 R	4.90 ± 0.01 ^a^	3.63 ± 0.00 ^a^	12.23 ± 0.01 ^a^
6.74 S	0.14 ± 0.00 ^b^	3.74 ± 0.03 ^b^	12.79 ± 0.06 ^a^
6.30 S	0.23 ± 0.02 ^a^	3.76 ± 0.05 ^b^	12.84 ± 0.07 ^a^
5.30 S	0.27 ± 0.01 ^a^	3.82 ± 0.01 ^a^	12.76 ± 0.02 ^a^
6.74 C	40.50 ± 0.80 ^a^	1.50 ± 0.01 ^c^	0.40 ± 0.01 ^a^
6.30 C	40.40 ± 1.02 ^b^	2.01 ± 0.02 ^a^	0.49 ± 0.02 ^a^
5.30 C	39.90 ± 1.01 ^b^	1.76 ± 0.03 ^b^	0.43 ± 0.01 ^a^

Data in the table represent the mean ± SD of three replicates; Different superscript letters among the same types of samples (raw milk, skim milk and cream) in the same column refer to statistically significant differences (*p* < 0.05); 6.74 R (raw milk pH 6.74), 6.30 R (raw milk pH 6.30), 5.30 R (raw milk pH 5.30), 6.74 C (cream pH 6.74), 6.30 C (cream pH 6.30), 5.30 C (cream pH 5.30), 6.74 S (skim milk pH 6.74), 6.30 S (skim milk pH 6.30) and 5.30 S (skim milk pH 5.30).

**Table 2:** Fat globule size, SSA, span and uniformity of raw milk, cream and skim milk samples with different pH.

Samples	Dv(10) (μm)	Dv(50) (μm)	Dv(90) (μm)	Specific Surface Area (cm^2^/g)	Span	Uniformity
6.74 R	0.03 ± 0.00 ^e^	0.65 ± 0.21 ^f^	7.65 ± 0.06 ^e^	65.41 ± 1.06 ^c^	5.51 ± 0.81 ^d^	2.14 ± 0.23 ^b^
6.30 R	0.04 ± 0.00 ^e^	2.52 ± 0.19 ^e^	12.37 ± 2.13 ^d^	56.96 ± 2.03 ^d^	6.41 ± 0.55 ^c^	2.49 ± 0.11 ^b^
5.30 R	0.04 ± 0.00 ^e^	2.96 ± 0.20 ^e^	10.53 ± 0.07 ^c^	47.68 ± 4.11 ^e^	8.05 ± 0.29 ^b^	4.41 ± 0.30 ^a^
6.74 C	2.16 ± 0.00 ^a^	8.93 ± 0.11 ^a^	51.42 ± 0.73 ^a^	1.69 ± 0.13 ^h^	3.62 ± 0.31 ^d^	1.67 ± 0.01 ^d^
6.30 C	1.57 ± 0.02 ^b^	7.28 ± 0.10 ^b^	48.21 ± 2.10 ^b^	2.04 ± 0.05 ^g^	4.88 ± 0.20 ^e^	1.78 ± 0.09 ^d^
5.30 C	0.97 ± 0.04 ^c^	5.86 ± 0.26 ^c^	48.14 ± 8.50 ^b^	2.28 ± 0.02 ^g^	12.65 ± 0.41 ^a^	2.43 ± 0.25 ^b^
6.74 S	0.02 ± 0.00 ^e^	0.07 ± 0.01 ^g^	0.25 ± 0.01 ^f^	110.10 ± 2.35 ^a^	3.06 ± 0.06 ^f^	1.97 ± 0.24 ^c^
6.30 S	0.02 ± 0.00 ^e^	0.08 ± 0.02 ^g^	0.28 ± 0.00 ^f^	103.62 ± 10.24 ^b^	3.15 ± 0.13 ^f^	2.57 ± 0.07 ^b^
5.30 S	0.81 ± 0.02 ^d^	5.29 ± 0.52 ^d^	9.18 ± 0.24 ^c^	8.32 ± 0.21 ^f^	1.59 ± 0.08 ^g^	0.43 ± 0.00 ^e^

Data in the table represent the mean ± SD of three replicates; Different superscript letters in the same column refer to statistically significant differences (*p* < 0.05); 6.74 R (raw milk pH 6.74), 6.30 R (raw milk pH 6.30), 5.30 R (raw milk pH 5.30), 6.74 C (cream pH 6.74), 6.30 C (cream pH 6.30), 5.30 C (cream pH 5.30), 6.74 S (skim milk pH 6.74), 6.30 S (skim milk pH 6.30) and 5.30 S (skim milk pH 5.30).

## Data Availability

Data generated during the study are available from the corresponding author upon request.
